# Hepatitis C Virus Adaptation to T-Cell Immune Pressure

**DOI:** 10.1155/2013/673240

**Published:** 2013-03-11

**Authors:** A. Plauzolles, M. Lucas, S. Gaudieri

**Affiliations:** ^1^Centre for Forensic Science, University of Western Australia, Nedlands, WA 6009, Australia; ^2^Institute for Immunology and Infectious Diseases, Murdoch University, Murdoch, WA 6050, Australia; ^3^School of Anatomy, Physiology and Human Biology, University of Western Australia, Nedlands, WA 6009, Australia

## Abstract

Replication of the hepatitis C virus (HCV) is an error-prone process. This high error rate results in the emergence of viral populations (quasispecies) within hosts and contributes to interhost variability. Numerous studies have demonstrated that both viral and host factors contribute to this viral diversity, which can ultimately affect disease outcome. As the host's immune response is an important correlate of infection outcome for HCV, many of these viral variations are strongly influenced by T-cell immune pressure and accordingly constitute an efficient strategy to subvert such pressures (viral adaptations). This paper will review the data on viral diversity observed between and within hosts infected with HCV from the acute to the chronic stage of infection and will focus on viral adaptation to the host's T-cell immune response.

## 1. Hepatitis C: A Major Global Health Issue

Hepatitis C is one of the most common blood-borne diseases in the world with estimated 150 million individuals infected with HCV worldwide (~3% of the world's population) [1]. As such, HCV has become a serious global health problem as a significant proportion of individuals with HCV infection progress to end-stage liver disease (more than 350,000 people die every year from hepatitis-C-related liver diseases) and is a major cause for liver transplantations in the world.

With a high replication rate, HCV can produce approximately 10^10^ to 10^12^ virions per day within an infected individual [2, 3] resulting in the generation of a large number of viral variants (quasispecies) with an error rate estimated to be between 10^−3^ and 10^−5^ mutations per nucleotide per genomic replication [4]. The quasispecies population within an infected individual is therefore composed of a pool of closely related viral variants that are present at different frequencies depending on their replication efficiency or fitness in the host's environment. Such diversity constitutes a real challenge to the immune responses developed by the host to fight against such pathogens as well as impedes the development of an effective vaccine or new treatments.

At present, no vaccine has been developed for HCV and current treatment, which consists of a combination of pegylated interferon alpha and ribavirin, remains expensive, has serious side effects, and is only moderately effective with about 50–60% of subjects reaching a sustained virological response (SVR) (reviewed in [5–7])). New direct acting anti-HCV drugs have recently been approved for clinical use by various governing bodies worldwide and result in much higher rates of sustained viral clearance, particularly for the common HCV genotype 1 strain. However, these new anti-HCV drugs can select for drug resistance mutations and therapy does not stop reinfection, a common event in at-risk populations such as prisoners and intravenous drug users. The development of an effective vaccine against HCV is still a priority but further research into understanding the host-viral interaction is necessary.

## 2. Determinants of the HCV Infection Outcome

Of those individuals infected with HCV about 70% develop chronic or persistent infection. The reason why some individuals manage to eliminate the virus during the acute phase of the infection (referred to as spontaneous resolvers) while others do not is still not entirely clear; however, the host's immune response is an important correlate of infection outcome. In support of this, studies have revealed that genetic variations in genes that are known to be involved in both the innate and adaptive immune response are associated with infection outcome (reviewed in [8]). Specifically, variations within the Human Leucocyte Antigen (HLA) genes, NK cell receptors such as the Killer Immunoglobulin-like receptors (KIRs), chemokines, interleukins, interferons, and interferon-stimulated genes have been associated with HCV infection outcome. However, aside from the recent genome-wide association studies (GWAS) highlighting *IL-28B* variants as the strongest host genetic predictor of infection outcome [9–12], these studies are typically limited by low subject numbers and lack of subjects from different ethnic populations (with different allelic distributions for many of these polymorphic genes) as well as a restricted number of cohorts that include acute HCV-infected individuals (a critical phase of the infection), and accordingly many genetic associations with infection outcome are rarely confirmed between cohorts. More importantly, given the close relationship between the host and virus, few studies have accounted for host and viral diversity in the same study. It is likely that the host's immune response and the adaptive potential of the incoming virus and subsequent viral adaptations during the acute phase of infection will influence infection outcome [13, 14]. Thus, the host's immune response has been identified as one of the most important selective pressures that shapes the quasispecies population [15, 16].

## 3. Effect of Host T-Cell Immune Responses on HCV Diversity

The immune system is a network of biological structures and processes aimed at protecting the human body against foreign particles that threaten its integrity or proper functioning. While innate immune responses do not target the virus in a specific manner, they do provide an immediate reaction that can slow down viral replication. On the contrary, the adaptive immune response including both humoral (e.g., antibodies) and cellular (e.g., CD4^+^ and CD8^+^ T cells) responses specifically targets HCV particles and infected cells and is generally detectable within a few weeks after the entry of the pathogen into the organism. Strong, sustained and multispecific CD4^+^ and CD8^+^ T-cell responses have both been associated with self-limited HCV infection in humans and chimpanzees [16–19]. Given the specificity of the cellular immune response, we focus here on how T-cell responses affect viral diversity.

### 3.1. Influence of Adaptive Immune Responses on HCV Diversity: Viral Adaptation to HLA-Restricted T-Cell Responses

HLA genes are the most variable loci in the genome with several hundreds of different alleles found within populations across the world. As such, the host's HLA repertoire is a major determining factor in the specificity of the adaptive immune response to HCV. Individuals are able to present varying viral peptides to the host's immune response thus allowing the host to differentiate self and non-self (including pathogens). HLA Class I (HLA-A, HLA-B, and HLA-C) proteins present peptides to CD8^+^ T cells, and HLA Class II (HLA-DR, HLA-DQ) proteins present peptides to CD4^+^ T cells. However, the efficacy of the host's virus-specific T-cell response is compromised by the mutability of the viral genome within HLA-restricted T-cell epitopes and this represents an important escape strategy of HCV [20, 21]. Accordingly, it would be expected that heterozygosity at the HLA loci would be advantageous for the host and studies have indeed shown that HLA heterozygosity is associated with good outcome following infection with HCV [22] as has also been shown for another highly mutable pathogen, HIV [23].

### 3.2. HCV Adaptation within CD8^+^ T-Cell Epitopes

The relevance of HCV immune escape mutations in infection outcome has been demonstrated in both humans [21, 24, 25] and chimpanzees [26–28] (reviewed in [29]). However, some of the most informative studies highlighting the influence of the host's immune response on HCV sequence and subsequently on infection outcome in humans have come from single source outbreaks. In these studies, specific HLA alleles have been associated with infection outcome [30], and the presence or lack of particular HLA-associated escape mutations in the source sequence reflects some of the HLA alleles associated with infection outcome in these cohorts [13]. For example, in the Irish single source cohort in which a large number of women in the late 1970s were infected with HCV through the administration of anti-D-immunoglobulin that had been contaminated with an HCV genotype 1b strain originating from a single infected individual [31], the HLA Class I allele HLA-B*08 is associated with poor outcome [13]. Subsequent analysis of the source HCV sequence has shown that the strain had a known escape mutation within an immunodominant HLA-B*08 T-cell epitope in NS3 that may have affected the ability of new hosts that expressed HLA-B*08 to present this important T-cell target [13, 32]. This mutation was, for the most part, retained in HLA-B*08 positive subjects with chronic HCV infection but had reverted in most HLA-B*08 negative subjects. Furthermore, in the same cohort, the HLA Class I allele HLA-A*03 was shown to be protective [30] and subsequent sequence analysis of HCV sequences from subjects in the cohort showed that most HLA-A*03 individuals who went on to develop chronic infection carried a small number of escape mutations in HLA-A*03-restricted T-cell targets that may have facilitated the persistence of the virus [13, 33].

In the late 70s, another large single source HCV outbreak similar to the HCV outbreak in Ireland occurred in Germany after inoculation of contaminated anti-D-immunoglobulin. Although both cohorts involve a single source HCV genotype 1b, the findings between the two cohorts show noticeable differences. While the study of the Irish cohort has shown to be very informative through the identification of protective alleles as well as unfavourable alleles (i.e., HLA-B*08, HLA-A*03), the German cohort does not confirm these associations. In their study, Ziegler et al. (involving the German cohort) [14] identified HLA-A*31 as the only allele showing a significantly higher frequency among spontaneous resolvers while no other HLA alleles were associated with infection outcome. Furthermore, Ziegler et al. [14] suggest that sequence differences within key HCV epitopes in the viral source may explain the dissimilarities observed between the two single source cohorts (German and Irish cohorts). Accordingly, their analysis revealed the presence of substitutions in the source virus that were likely to have altered the selection pressure and ultimately viral evolution within the German cohort.

### 3.3. Suggested HCV Adaptation within CD4^+^ T-Cell Epitopes

Numerous studies have shown an association between HLA Class II alleles and HCV infection outcome [8, 27, 34, 35]. The HLA-DRB1*0701 allele has previously been associated with persistence [36] while other studies have shown a strong association between HLA-DRB1*0101, HLA-DRB1*1101, and HLA-DQB1*0301 and viral clearance [37–39]. Furthermore, studies on HCV using the chimpanzee model and humans have identified several CD4^+^ T-cell epitopes targeted within subjects experiencing viral resolution [34, 40–42]. However, although there are many studies that have documented immune escape within CD8^+^ T-cell epitopes via the emergence of mutations, there are few studies that examine the implications of viral variation on CD4^+^T-cell responses. One such study, using synthesised peptides, showed that viral variants failed to stimulate proliferation or cytokine production by CD4^+^ T cells [43–45], suggesting that the emergence of mutations may be an immune escape strategy. Similarly, another study showed that the presence of naturally occurring amino acid substitutions in an HLA-DRB1*15-restricted T-cell epitope in the NS3 protein failed to stimulate proliferation [46]. In support of these findings, another study involving chimpanzees challenged with a clonal HCV presenting the same sequence as the antigens used for immunization showed viral resolution in two chimpanzees while the third animal only experienced transient control of the virus with viral rebound that correlated with the emergence of mutations within the NS3 and NS5A regions; these mutations appeared to impair CD4^+^ T-cell recognition [47]. Such studies suggest the relevance of immune escape within CD4^+^ T-cell epitopes; however, further studies on specific immune escape mutations are limited, possibly hampered by the more promiscuous binding properties of HLA Class II peptide pockets and limited HLA Class II peptide binding prediction programs. Given the importance of CD4^+^ T-cell responses in HCV infection outcome, there is a need for better understanding of the dynamics and relevance of CD4^+^ T-cell escape in HCV.

### 3.4. HCV Adaptation at the Population Level

The host's immune response can not only shape the viral population during the course of infection within a single host but also the viral species circulating within the human population. As the host HLA molecules regulate immune responses by presenting specific viral epitopes to T cells and viral polymorphisms within or flanking these epitopes can compromise the efficacy of these T-cell responses, then viral adaptations should be identified at the population level as HLA-specific HCV polymorphisms. Genetic-based studies that have sampled the host HLA and viral diversity of hundreds of chronic infected individuals for both HCV and HIV have identified such associations between specific viral polymorphisms and the HLA types of individuals within a host population that mark true in vivo T-cell targets [21, 48–50]. Furthermore, immune escape mutations with negligible fitness cost associated with common HLA alleles (or offset with compensatory mutations) may remain in the transmitted virus and be propagated in the population resulting in it becoming the wildtype [50–53], an important consideration in the design of a T-cell-based vaccine.

However, it is unclear how much HCV genetic diversity is due to the host's immune response as HCV evolution is likely to be shaped by evolutionary forces that include genetic drift and both positive and purifying selection pressures [54, 55]. Using a single source outbreak again, Merani et al. [13] showed that of the number of sites in the new hosts that differed from the source sequence (sampled about 20 years later) only a proportion (<20%) could be attributed to HLA-restricted T-cell immune pressure. Interestingly, some of these putative HCV escape mutations were linked to other variants elsewhere in the genome possibly representing compensatory mutations suggesting limited plasticity in the virus [13].

Overall, HCV escape from HLA-restricted T-cell responses can confer a selective advantage to the virus resulting in the fixation (or dominance) of specific strains within the host and to some extent will drive HCV evolution at the population level.

### 3.5. HCV Adaptation during the Early Phase of Infection

As discussed above, HCV adaptation within T-cell epitopes can lead to escape from these immune responses and has been shown to be associated with viral persistence [24, 25]. However, although the host immune responses constitute a growth restraint that will shape the quasispecies population within a host, our understanding of the dynamics of viral adaptation during the critical acute phase of HCV infection is relatively limited due to the lack of large acute HCV infection cohorts and the small number of known HCV T-cell epitopes.

Previous studies examining viral adaptation during acute HCV infection on a small number of subjects showed that the proportion of mutations likely to be associated with CD8^+^ T-cell immune pressure was 25% or less [24, 55]. These results contrast with those from acute HIV-1 and SIV infection studies, which suggest that the proportion of viral adaptations associated with CD8^+^ T-cell immune pressure can be greater than 50% [56–58]. However, these earlier comparisons were hampered by the discrepancy in the number of known T-cell epitopes for the two viruses in that there are a greater number of known T-cell epitopes that cover the HIV proteome compared to the HCV proteome (http://www.hcv.lanl.gov; http://www.immuneepitope.org).

In a recent study of 21 acute HCV-infected individuals from Germany, Pfafferott et al. (2011) attempted to redress this issue by including HLA-associated viral polymorphisms generated from earlier genetic studies and compared these results to data from another study on 98 acute HIV-infected individuals using similar methodology [15]. The analysis from this study revealed a higher proportion of mutations associated with T-cell pressure in HIV (30–60%) than that for HCV (31.8%) with little evidence of early reversion for HCV in comparison to the substantial propensity of HIV to revert from an adapted form to its wildtype form when the selecting immune pressure is no longer applied [56]. These differences suggest that HCV presents a lower tolerance toward nucleotide substitution in comparison to HIV with HCV subject to functional constraints that limit its plasticity and potentially its capacity to escape T-cell responses.

### 3.6. Immune Escape Patterns: Reversion and Compensatory Mutations

Mutations associated with a high fitness cost may be either incompatible with viral viability and consequently will not be observed or can be compensated by the emergence of more mutation(s) that limit the deleterious effect of the first variation(s). It is important to consider a mutation not only as a single change but also as variations along the genome that can be related (e.g., compensatory mutations). Interactions between mutations have been studied [59] revealing a combined effect rather than a simple sum of individual effects [60]. This suggests an epistasis between mutations where the effect of a mutation can result or be the result of one or more mutations. HCV has the capability to change within a host during the course of infection, especially during the acute phase as the virus has to adapt to a new host including likely new immune pressures. If the fitness cost of such changes is too high, the emergence of compensatory mutation(s) that limit the fitness cost of the first variation(s) is likely to be observed, as has been reported for HIV [51, 61, 62].

Some HCV escape mutations have been shown to have a fitness cost. A recent study demonstrated the fitness cost associated with mutations within the immunodominant HLA-B*08-restricted epitope in NS3 reducing the viral replication capacity to 60% [63]. Similarly, another study showed the impact on fitness of an escape mutation within an HLA-A*03-restricted epitope in NS3 [33]. This latter study also showed that another mutation within the same T-cell epitope offsets the fitness cost associated with the immune escape mutation. Furthermore, studies have demonstrated that the mechanism of immune escape can either be a single or multistep process. Indeed, a single mutation if located within a peptide-HLA binding site might strongly impair the mechanisms of defence directed against HCV while another mutation situated in a site that is functionally less relevant might not have enough impact by itself to subvert the immune mechanism pressurising the virus and may therefore require the support of other mutation(s) to efficiently impair the immune response or to compensate the viral fitness cost caused by the previous mutation. Consequently, the mechanism leading to viral immune escape in HCV can require clustered mutations in order to reach a balance between an acceptable viral fitness and an efficient immune escape [64, 65]. The study on the immunodominant HLA-B*27-restricted T-cell epitope in NS5B, which has previously been associated with protection [66] for the HCV genotype 1 strain, revealed that this T-cell epitope requires multiple substitutions in order to escape the immune response [64, 67]. Data have shown that mutations within this T-cell epitope are observed within chronic HCV-infected individuals that are HLA-B27 negative and maintained during the course of the infection thus increasing the opportunity to present adapted variants with all mutations necessary to bypass the immune cells targeting this viral region [64]. These results also suggest lack of reversion in HCV.

The phenomenon of reversion to the wildtype is generally observed when the pressure that is selected for the initial mutation(s) is no longer applied. The mechanism of reversion was initially observed in HIV infection [51, 61, 62] and subsequently in HCV infection [21, 24, 32]. However, an absence of reversion despite the absence of the selective pressure can be partly explained by the emergence of compensatory mutations. A recent study demonstrated that the phenomenon of reversion along the HCV genome was limited, with only a minor proportion of mutations occurring within T-cell epitopes during early infection that were restricted by HLA alleles that were not present in the new host [15]. This absence of reversion suggests a limited or no fitness cost associated with these escape mutations, or if fitness cost resulted from these mutations, it must have been compensated by other substitutions. The lack of reversion for HCV raises the concern that adapted viral variants presenting advantageous mutations toward immune T-cell responses may circulate in the population and therefore facilitate escape from protective immune responses.

An analysis for covarying sites within the HCV genome was performed for the chronic HCV infected subjects in the Irish single source cohort [13]. In that study, significant covariations (that differed from source) occurred locally within the same protein and a number of sites were linked to sites more distantly positioned within the genome. As expected, several of these sites were putative viral adaptation sites. Co-variations, which become fixed across the HCV genome, may restrict the ability of HCV to revert upon entering a new non-HLA matched host.

In order to examine the plasticity of the HCV genome, an examination of the pattern of substitution observed for the virus is necessary.

## 4. Pattern of HCV Substitution Suggests Limited Plasticity of the Virus

The analysis of substitutions along the viral genome allows one to evaluate viral evolution with synonymous mutations that are considered neutral (assuming that silent mutations are not subject to selection pressure—although there has been some suggestion of quasi selection at synonymous sites relating to mutational pathways; [68]) and therefore reflect the mutational rate of the virus while nonsynonymous mutations are the result of mutation rate and selection pressures [69]. Accordingly, the proportion of non-synonymous substitutions per non-synonymous site (dN) to synonymous substitutions per synonymous site (dS) is a good estimation of the selective pressures acting on the virus in specific regions and even at single sites [70–72].

The number of synonymous and nonsynonymous changes across the genome was examined for the viruses in chronic HCV-infected individuals from the Irish single source cohort [13]. An abundance of synonymous changes was observed indicating that purifying selection was likely to be occurring that will to some extent limit the plasticity of HCV. Using a large dataset of HCV sequences from chronic HCV-infected individuals from Australia, Switzerland, and the UK, Rauch et al. (2009) found that most codons in the nonstructural proteins were under purifying selection (66%) with only 1.4% showing evidence for positive selection. Pfafferott et al. (2011) also found a similar pattern in the evolution of HCV during early infection.

## 5. New High-Resolution Next Generation Sequencing Techniques to Improve Understanding of Host/Viral Interactions

Although viral diversity remains a good indicator of viral evolution, clonal sequence analysis remains limited and low frequency (<20%) quasispecies are not able to be detected using the standard Sanger-based sequencing techniques. More sensitive sequencing techniques, such as next generation sequencing (NGS), are now on the market and represent a promising tool in the identification of low frequency mutations that enable escape from T-cell immune pressure. As such, NGS techniques are particularly useful to examine quasispecies changes over-time as it allows the identification of preexisting drug-resistant mutants or newly emerging escape mutations present at frequencies <1% among the quasispecies population [73].

Bull et al. (2011) analysed within-host evolutionary dynamics of HCV via the use of NGS and identified two bottleneck events during the acute phase of the infection suggesting strong selective pressures that limit viral diversity [74]. The first bottleneck refers to the transmission event, which consists of the transfer of an inoculum of viruses from donor to recipient. In this study, it was demonstrated that among the numerous viral variants transmitted, only one to two viral variants successfully establish the new infection. The second bottleneck was identified 100 days post-infection with a decrease in viral diversity, which was likely associated with adaptive immune responses targeting the virus (temporal proximity to seroconversion) as this second bottleneck was followed by clearance or emergence of new variants leading to chronicity with mutations occurring in known CD8^+^ T-cell epitopes. These two bottlenecks were observed within both spontaneous resolvers and chronic evolvers thus suggesting a common evolutionary pattern during the acute phase of the infection. In addition, data revealed a limited emergence of substitutions and viral variants during the acute phase giving the high mutation rate of HCV (with a majority of substitutions occurring at low frequencies (below 2%)), which is consistent with Pfafferott et al.'s 2011 [15]. However, further studies are required in order to determine if this limited variability is due to selective immune pressure from the host and/or fitness cost from the virus.

Such studies using NGS will therefore be necessary in the future to acquire a better understanding of the host-viral dynamics in differing environmental (immune and drug) contexts and will be important in vaccine design and in the development of new antiviral drugs.

## 6. Conclusion

HCV adaptation is an important immune escape strategy used by the virus and is a likely correlate of infection outcome. However, although HCV has a high mutation rate, the viral genome does appear to exhibit restricted plasticity as evidenced by an accumulation of silent changes. Furthermore, there does appear to be a lack of reversion in HCV in the absence of the original immune pressure, and this suggests that there is stabilisation via compensatory mutations particularly giving the reported fitness cost of various escape mutations, the detection of compensatory mutations within epitopes that offset the fitness cost of the escape mutation, and evidence of co-variation within and between HCV proteins. If reversion of an adapted form of the virus to its wildtype is limited with HCV, this raises the concern about the increase in resistant variants (resistance toward drugs or common HLA alleles) circulating within infected populations.
